# Protective Effects of Anti-IL17 on Acute Lung Injury Induced by LPS in Mice

**DOI:** 10.3389/fphar.2018.01021

**Published:** 2018-10-04

**Authors:** Renato Fraga Righetti, Tabata Maruyama dos Santos, Leandro do Nascimento Camargo, Luciana Ritha Cássia Rolim Barbosa Aristóteles, Silvia Fukuzaki, Flávia Castro Ribas de Souza, Fernanda Paula Roncon Santana, Marcus Vinicius Rodrigues de Agrela, Maysa Mariana Cruz, Maria Isabel Cardoso Alonso-Vale, Isabella Santos Genaro, Beatriz Mangueira Saraiva-Romanholo, Edna Aparecida Leick, Milton de Arruda Martins, Carla Máximo Prado, Iolanda de Fátima Lopes Calvo Tibério

**Affiliations:** ^1^Faculdade de Medicina FMUSP, Universidade de São Paulo, São Paulo, Brazil; ^2^Hospital Sírio-Libanês, São Paulo, Brazil; ^3^Universidade Federal de São Paulo, Instituto de Ciências Ambientais, Químicas e Farmacêuticas, Departamento de Ciências Biológicas, Diadema, São Paulo, Brazil; ^4^Public Employee of São Paulo Hospital (IAMSPE), São Paulo, Brazil; ^5^University of City of São Paulo (UNICID), São Paulo, Brazil; ^6^Department of Bioscience, Federal University of São Paulo, Santos, São Paulo, Brazil

**Keywords:** acute lung injury, inflammation mediators, interleukin 17, remodeling, oxidative stress

## Abstract

**Introduction:** T helper 17 (Th17) has been implicated in a variety of inflammatory lung and immune system diseases. However, little is known about the expression and biological role of IL-17 in acute lung injury (ALI). We investigated the mechanisms involved in the effect of anti-IL17 in a model of lipopolysaccharide (LPS)-induced acute lung injury (ALI) in mice.

**Methods:** Mice were pre-treated with anti-IL17, 1h before saline/LPS intratracheal administration alongside non-treated controls and levels of exhaled nitric oxide (eNO), cytokine expression, extracellular matrix remodeling and oxidative stress, as well as immune cell counts in bronchoalveolar lavage fluid (BALF), and respiratory mechanics were assessed in lung tissue.

**Results:** LPS instillation led to an increase in multiple cytokines, proteases, nuclear factor-κB, and Forkhead box P3 (FOXP3), eNO and regulators of the actomyosin cytoskeleton, the number of CD4+ and iNOS-positive cells as well as the number of neutrophils and macrophages in BALF, resistance and elastance of the respiratory system, ARG-1 gene expression, collagen fibers, and actin and 8-iso-PGF2α volume fractions. Pre-treatment with anti-IL17 led to a significant reduction in the level of all assessed factors.

**Conclusions:** Anti-IL17 can protect the lungs from the inflammatory effects of LPS-induced ALI, primarily mediated by the reduced expression of cytokines and oxidative stress. This suggests that further studies using anti-IL17 in a treatment regime would be highly worthwhile.

## Introduction

Acute lung injury (ALI) and acute respiratory distress syndrome (ARDS) can result from various pathologies including sepsis, microbial infection, trauma or ischemia/reperfusion, and can lead to acute respiratory failure with a mortality rate of approximately 40% (Modrykamien and Gupta, 2015). The pathogenesis of ALI/ARDS is characterized by epithelial integrity disruption and the infiltration of polymorphonuclear cells (PMNs) into the lungs, which may cause interstitial edema and alveolar collapse (Castillo et al., 2015). In addition, macrophages and neutrophils have been shown to release pro-inflammatory cytokines such as interleukin (IL)-1β, IL-6, IL-8, and tumor necrosis factor (TNF)-α and as well as profibrotic growth factors such as platelet-derived growth factor, transforming growth factor beta (TGF-β; Pinheiro et al., 2015; Blondonnet et al., 2016). Thus, macrophages and neutrophils may be important mediators in initiating the inflammatory response, regulating fibroblast function and oxidative stress in the later stage of ALI/ARDS (Pinheiro et al., 2015; Blondonnet et al., 2016).

Nitric oxide (NO) produced in large amounts by inducible nitric oxide synthase (iNOS) occurs mainly in macrophages inflammatory situations and causes lipid peroxidation and the generation of isoprostane (Villegas et al., 2014). The 8-isoprostane prostaglandin 2α (8-iso-PGF2α) is considered the predominant marker of tissue oxidative damage form generated during free radical attack of cell membranes (Elfsmark et al., 2018). Exhaled NO (eNO) has been used as a marker of both inflammation and oxidative stress (Boshier et al., 2013).

The nuclear transcription factor kB (NFkB) is a transcription factor found in the cytoplasm. The increase activation of NFkB in alveolar macrophages of patients with established ALI/ARDS, resulting in pro-inflammatory cytokines increase, such as TNF-a, IL-1β, IL-6, IL-8, and oxidative stress activation (Bittencourt-Mernak et al., 2017). The continuous inflammation and activation of matrix metalloproteinase (MMP) 9 and 12 and tissue inhibitor of metalloproteinase 1 (TIMP-1) can be followed by alterations in the lung extracellular matrix, which can occur earlier in ARDS and contribute to decreased lung function (González-López and Albaiceta, 2012; Bittencourt-Mernak et al., 2017).

Rho-kinase (ROCK) is a small GTPase and a major downstream effector of RhoA, ROCK promotes the modulating stress fiber formation, actin cytoskeleton organization, and smooth muscle cell contraction (Pigati et al., 2015). Two mammalian ROCK homologs have been identified, ROCK 1 and ROCK 2 (Defert and Boland, 2017).

T-helper (Th)-17 are a unique subset of cluster of differentiation 4 (CD4+) T-helper cells characterized by the production of pro-inflammatory IL-17A, IL-17F, and IL-22 and the their differentiation is mediated by the activation of T helper cells in the presence of a combination of TGF-β, IL-6, IL-1β, and IL-23 (Ghoreschi et al., 2010). IL-6 combined with TGF-β induces the development of Th17 effector cells. IL-17 acts by activating iNOS and inducing the expression of macrophage, IL-1β, IL-6, IL-8, TNF-α, and several chemokines, which collaborate to potentiate the inflammatory process (Gonçalves-de-Albuquerque et al., 2017).

Th17 has been implicated in the development or pathogenesis of several autoimmune and inflammatory diseases (Sakaguchi et al., 2016), including those of the lung, such as cystic fibrosis, asthma, chronic obstructive pulmonary disease and granulomatous disease (Hsu et al., 2016; Yanagisawa et al., 2017, O'Dwyer et al., 2016; Hasegawa et al., 2017). However, IL-17 also plays an important role in protective immunity against some bacterial infections, mainly through the recruitment and activation of neutrophils. Thus, the regulation of Th-17 is important in that either an excess or deficiency could result in a modulation of these diseases (Ding et al., 2017).

Mikacenic et al. (2016) shows elevated circulating and alveolar levels of IL-17 in human with ARDS, but the role of the Th-17 immune response in ALI/ARDS has not been defined. Therefore, in this study we evaluated the effect and mechanisms involved in after pretreatment with anti-IL-17 in the early phase of a mice model of ALI induced by lipopolysaccharide (LPS). Specifically we measured changes in the resistance and elastance of the respiratory system, multiple markers of inflammation, as well as oxidative stress and extracellular matrix remodeling in lung tissue. Understanding the role of IL-17 signaling in ALI/ARDS may contribute to the development of a future therapeutic target for this disease.

## Materials and methods

### Animals and experimental model

Thirty-two male BALB/c mice (25g to 30 g) were divided into four groups (*n* = 8 in each group):

E) SAL (saline solution-instilled),

F) LPS (LPS-instilled),

G) SAL-antiIL17 (saline solution-instilled and anti-IL17 treatment),

H) LPS-antiIL17 (LPS-instilled and anti-IL17 treatment).

All animals received humane care in accordance with the Guide for the Care and Use of Laboratory Animals (National Institutes of Health, 2011). This study was approved by the research committee of the School of Medicine at the University of São Paulo (São Paulo, Brazil) for animal studies (number 054/15).

### Anti-IL17 treatment

Anti-IL17 neutralizing antibody (R and D Systems, Abingdon, UK) was administered intraperitoneally at a dose of 7.5 μg/application, based on the protocol of Barlow et al. (2011) and Camargo et al. (2018), 1 h prior to intratracheal instillation of LPS/saline solution.

### Experimental model of ALI

For intratracheal instillation (I.T.) animals were anesthetized with isoflurane (2%), then a 1 cm-long midline cervical incision was made to expose the trachea, and LPS or saline was instilled using a bent 27-gauge tuberculin needle. LPS and LPS-antiIL17 groups received a single I.T. dose of Escherichia coli LPS (026: B6L3755, Sigma Aldrich, St. Louis, MO, USA) 5 mg.kg^−1^ suspended in saline solution (total volume = 0.05 mL per animal). SAL and SAL-antiIL17 groups received a single I.T. dose of 0.05 mL saline solution. After the procedure, the cervical incision was closed with a 5.0 silk suture, and the mice were returned to their cages (Bittencourt-Mernak et al., 2017).

### Exhaled nitric oxide (eNO)

Twenty-four hours after LPS or saline instillation, the animals were anesthetized using pentobarbital sodium (50 mg/kg i.p.), tracheostomized, connected to a Harvard 683 ventilator (Harvard Apparatus, South Natick, MA, USA) and mechanically ventilated at 60 breaths/min with a tidal volume of 10 mL/kg. A Mylar bag was connected at the expiratory port of the ventilator for 10 min by measured eNO levels and to avoid environmental contamination. This was then attached to the inspiratory breathing circuit input and NO filter. After sample collection, eNO concentrations were measured by chemiluminescence using a NOA 280 (Sievers Instruments Inc., Boulder, CO, USA). The analyzer was calibrated with an NO (nitric oxide) source certified at 47- parts per billion (ppb; White Martins, São Paulo, Brazil) and a zero NO filter (Sievers Instruments Inc.; Pigati et al., 2015).

### Respiratory system mechanics

An evaluation of respiratory system mechanics was conducted after the eNO measurement. A differential pressure transducer (Honeywell, Freeport, IL), pneumotachograph (Fleisch-4.0, OEM Medical, Richmond, VA, USA) and Honeywell 163PC01D36 differential pressure transducer were connected to the tracheal cannula to measure the tracheal pressure (Ptr) and airflow (V').

Lung volume (V) changes were determined by the digital integration of the airflow signal. Nine to ten respiratory cycles were averaged to provide one data point. The Ptr, V', and V signals were collected and stored on a microcomputer. Lung volume (V) changes were obtained by the electronic integration of V'. The respiratory system resistance (Rrs) and elastance (Ers) were computed by least squares fitting of the measured values of Ptr, V, and V. over 9 to 10 respiratory cycles using the following equation of motion of the respiratory system: Ptr (t) = Ers × V(t) + Rrs × V'. (t), where t is time. All data were collected and processed using LABDAT software (RHT-InfoData, Montreal, Quebec, Canada; Possa et al., 2012; Pigati et al., 2015).

### Bronchoalveolar lavage fluid (BALF) cell counts

After the mechanical respiratory systems evaluation, the animal's anterior chest wall was opened and it was euthanized by exsanguination *via* the abdominal aorta. The BALF was collected by flushing the lungs three times with 0.5 mL of 37°C sterile physiological saline (0.9% NaCl) via the tracheal cannula and the fluid samples were centrifuged at 420 × g at 4°C for 15 min. The cell pellets were resuspended in 1 mL of sterile physiological saline and 100 μL of each sample was cytocentrifuged in a Cytospin for 6 min at 450 rpm. The total cell counts were performed using a *Neubauer* chamber and for differential cells counts, cytospin slides were prepared and stained with *Diff-Quick* Reagent (Biochemical Sciences Inc., Swedesboro, NJ). 300 cells were counted per slide using an optical microscope, using the morphologic criteria (Vieira et al., 2007; Martins-Olivera et al., 2016).

### Lung histology

After BALF collection, the lungs were fixed dor 24 h in a sufficient volume of formalin (4%) to completely immerse the tissue, and then the lung was dehydrated in a solution of 70% ethanol, xylol, and afterwards, it was immersed in paraffin, and different regions of the lung were cut into slices to obtain 4-μm thick sections that were then mounted on to slides. The slides were stained with hematoxylin-Eosin and Picro-Sirius Red for analysis of collagen fibers, according to the following protocol:

Sections were deparaffinized, rinsed with water and hydrated in 95% alcohol before being stained with Picro-Sirius Red (Direct Red 80, C.I. 35780, Sigma Aldrich) at room temperature. Then, the slices were washed in running water for 5 min, and the sections were stained with Harris hematoxylin for 6 min and washed again in running water for 10 min. They were then dehydrated and mounted on slides (Possa et al., 2012). The histologic slides were not labeled with group information and the researcher had no knowledge of the procedures for each group.

### Immunohistochemistry

The expression of positive cells was evaluated with immunohistochemistry technique. The lung sections were deparaffinized and 0.3% hydrogen peroxide was applied for 35 min to inhibit endogenous peroxidase activity. Antigen retrieval was performed using citrate solution for 45 min to inhibit endogenous peroxidase activity (Possa et al., 2012).

The sections were incubated overnight at 4°C with anti-TNF-α (1:400; R&D Systems, Minneapolis, MN, USA), anti-IL1β (1:400), anti-IL6 (1:200), anti-IL8 (1:200), anti-IL10 (1:300), anti-IL17 (1:250), anti-CD4+ (1:600), anti-iNOS (1:400), anti-forkhead box P3 (FOXP3) (1:1200), anti-p65-NF-κB (1:250), anti-8-iso-PGF2α (1:1500), anti-TGF-β (1:600), anti-MMP-9 (1:500), anti-MMP-12 (1:450), anti-TIMP-1 (1:100), anti-actin (1:1600), anti- ROCK 1 (1:50), and anti-ROCK 2 (1:100; Santa Cruz Biotechnology Inc., Santa Cruz, CA, USA). The following day, the slides were washed in PBS and incubated with a secondary antibody using the ABCKit by Vectastain (Vector Elite-PK-6105 anti-goat), PK-6101 (anti-rabbit), and PK-6102 (anti-mouse). For visualization of positive cells, the slides were washed in PBS and proteins were visualized using 3,3'-diaminobenzidine chromosome (DAB; Sigma Chemical Co., St. Louis, MO, USA). Slide sections were contrasted with Harris hematoxylin (Merck, Darmstadt, Germany) and assembled using Entellan microscopy resin (Merck; Possa et al., 2012; Righetti et al., 2014).

### Quantification of collagen fibers, actin and 8-Iso-PGF2α volume fractions

We measured the total area of lung tissue and the area of collagen fibers, actin and 8-iso-PGF2α in 15 to 20 microscopic fields per lung using a 400x magnification and the Image-ProPlus 4.5 *Image Analysis System* (Media Cybernetics, Silver Spring, USA). The amount of collagen fibers, actin or 8-iso-PGF2α was expressed as a percentage based on the relationship between the quantity of collagen fibers, actin or 8-iso-PGF2α in a specific frame and the total area of that frame (volume fraction; Prado et al., 2006).

### Morphometric analysis

We assessed the density of inflammatory cells (mononuclear and polymorphonuclear cells) that were positive for CD4+, FOXP3, TNF-α, IL-1β, IL-6, IL-8, IL-10, IL-17, iNOS, p65-NFκB, TGF-β, MMP-9, MMP-12, TIMP-1, ROCK 1, and ROCK 2 in the lung tissue using conventional morphometry. Using a 100-point grid of a known area (62,500 μm2 at × 400 magnification) attached to the ocular of the microscope; we counted the number of points covering the lung tissue and the number of inflammatory positive cells in each field. The cell density was determined as the number of positive cells in each field divided by the tissue area and expressed as positive cells/10^4^μm^2^ (Weibel, 1979; Lanças et al., 2006; Righetti et al., 2014; Barreto do Carmo et al., 2016; Camargo et al., 2018). The morphometric measurements were performed on 15 fields per slices at ×1000 magnification.

### Evaluation of cytokines in lung tissue

Levels of TNF-α and IL-1β in the lung tissue were evaluated using ELISA in accordance with the manufacturer's instructions (Duo Set, R&D Systems, Minneapolis, MN, US) and previously used by (Bittencourt-Mernak et al., 2017).

### RNA extraction, reverse transcription and quantitative real-time PCR (RT-PCR)

The expression level of IL-6, IL-17, and arginase 1 (ARG-1) in the lungs was evaluated using real-time PCR (polymerase chain reaction) as previously reported by our group (Pinheiro et al., 2015). Expression of GAPDH was used as an internal control. The primer sequences were: GAPDH (5′-3′ sense: CCACCACCCTGTTGCTGTAG; 5′-3′antisense: CTTGGGCTACACTGAGGACC; 60°C; NM_008084) and IL-6 (5′-3′ sense: TTCTCTGGGAAATCGTGGAAA; 5′-3′ antisense: TCAGAATTGCCATTGCACAAC; NM_001314054); IL-17 (5′-3′ sense: TGAAGGTCAACCTCAAAGTCT, 5′-3′ antisense: GAGGGATATCTATCAGGGTCTTCAT) and ARG-1(5′-3′ sense GCACTCATGGAAGTACACGAGGAC, 5′-3′ antisense: CCAACCCAGTGATCTTGACTGA). The results were obtained as the cycle number at which logarithmic PCR plots cross a calculated threshold line and used to determine ΔCt values [ΔCt = (Ct of the target gene)–(Ct of the house-keeping gene)]. The results are expressed as arbitrary units using the transformation: Expression = 1000 × (2^−Δ*ct*^) arbitrary units (Camargo et al., 2018).

### Alveolar collapse

The volume fraction of the lung occupied by collapsed alveoli (alveoli with rough or plicate walls) or normal pulmonary areas (those not exhibiting over distended or plicate walls) were determined by the point-counting technique (Weibel, 1963) at a magnification of ×200 across 10 random, non-coincident microscopic fields (Riva et al., 2008; Saddy et al., 2010).

### Data analysis

All analyzes were conducted using SigmaPlot 11.0 software (Systat Software, SPSS Inc., USA). Results are represented as the means ± SE and One-Way Analysis of Variance (ANOVA) followed by the Holm-Sidak method for multiple comparisons. A *p*-value < 0.05 was considered statistically significant.

## Results

### Respiratory mechanics

**Figure 1:**
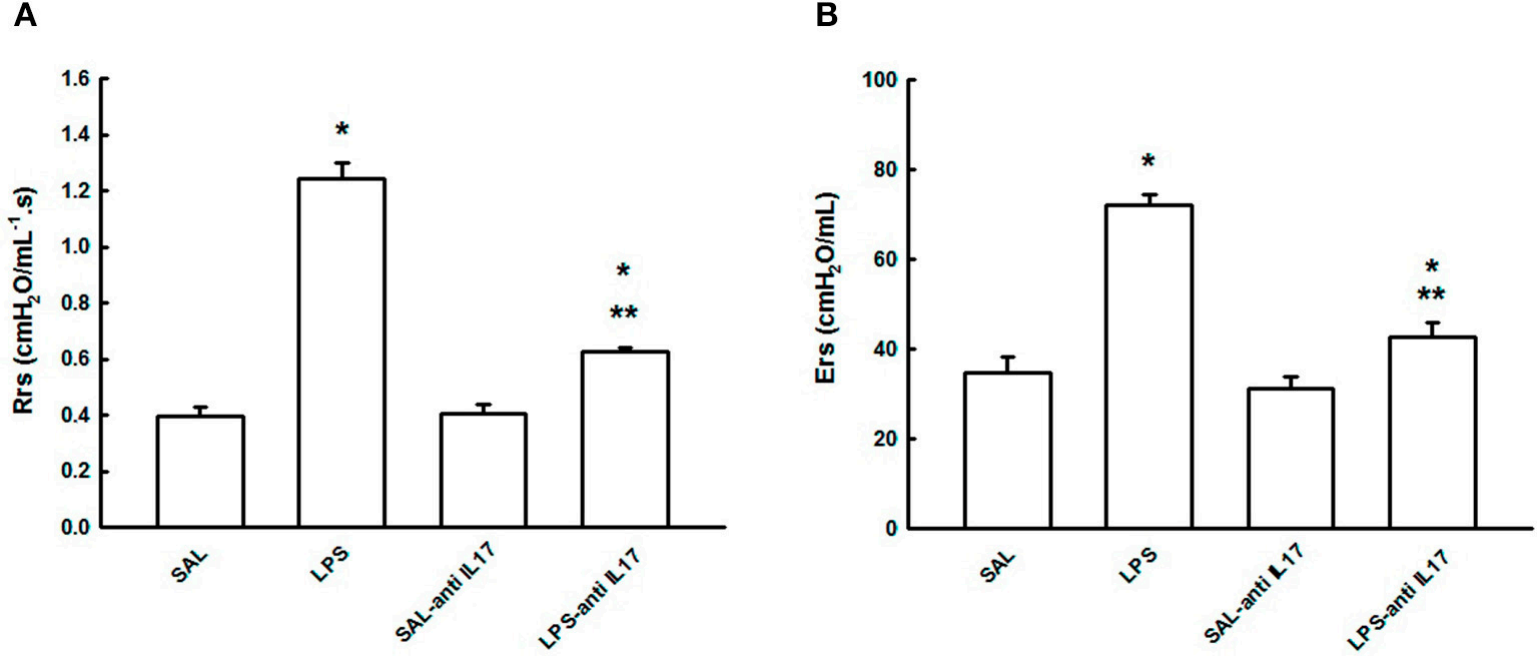
Effects of the pretreatment with anti-IL17 on mechanical evaluation: **(A)** Respiratory system resistance and **(B)** Respiratory system elastance. *P < 0.05 compared with SAL and SAL-antiIL17 groups; **P < 0.05 compared to the LPS group.

Figure 1 shows the functional consequences of pretreatment with anti-IL17 in this animal model of acute ling injury on Rrs (A) and Ers (B) in the four experimental groups. LPS instillation increased both pulmonary Rrs and Ers compared with the SAL and SAL-antiIL17 groups (*P* < 0.05), which was decreased by anti-IL17 in both cases (*P* < 0.05).

### Exhaled nitric oxide

**Figure 2:**
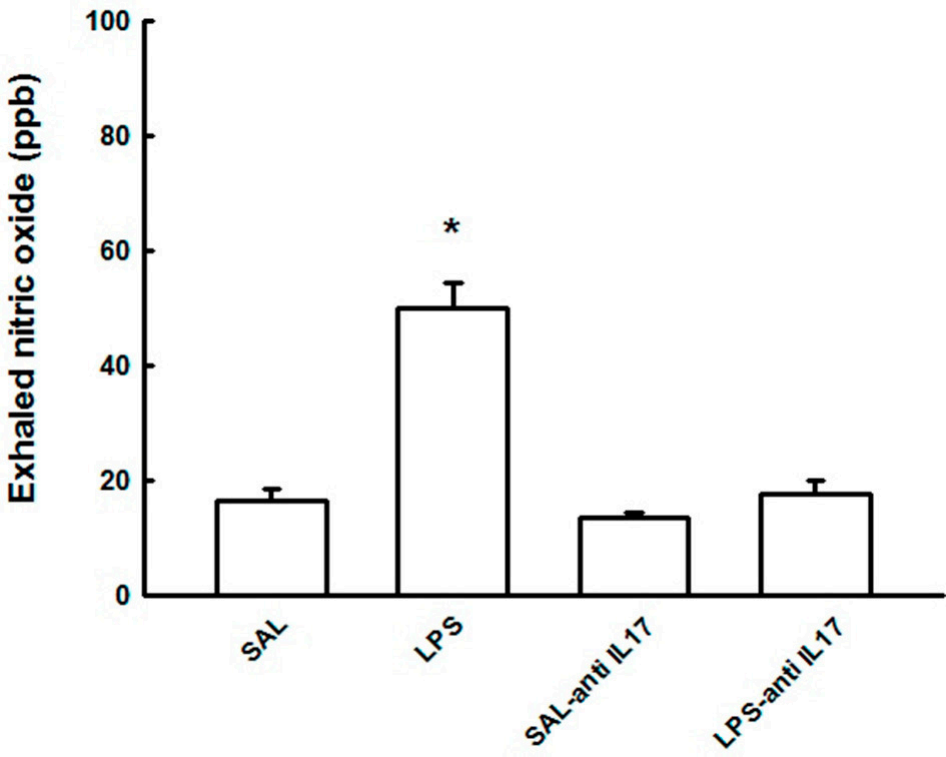
Effects of the pretreatment with anti-IL17 on exhaled nitric oxide (ppb). *P < 0.05 compared to the SAL, SAL-antiIL17 and LPS-antiIL17 groups.

Figure 2 shows the effect of the pretreatment with anti-IL17 on the pro-inflammatory concentration of eNO in the four experimental groups. LPS instillation increased eNOS concentration compared to the SAL group and SAL-antiIL17 groups (*P* < 0.05), which was decreased by anti-IL17 (*P* < 0.05).

### BALF cell counts

**Figure 3:**
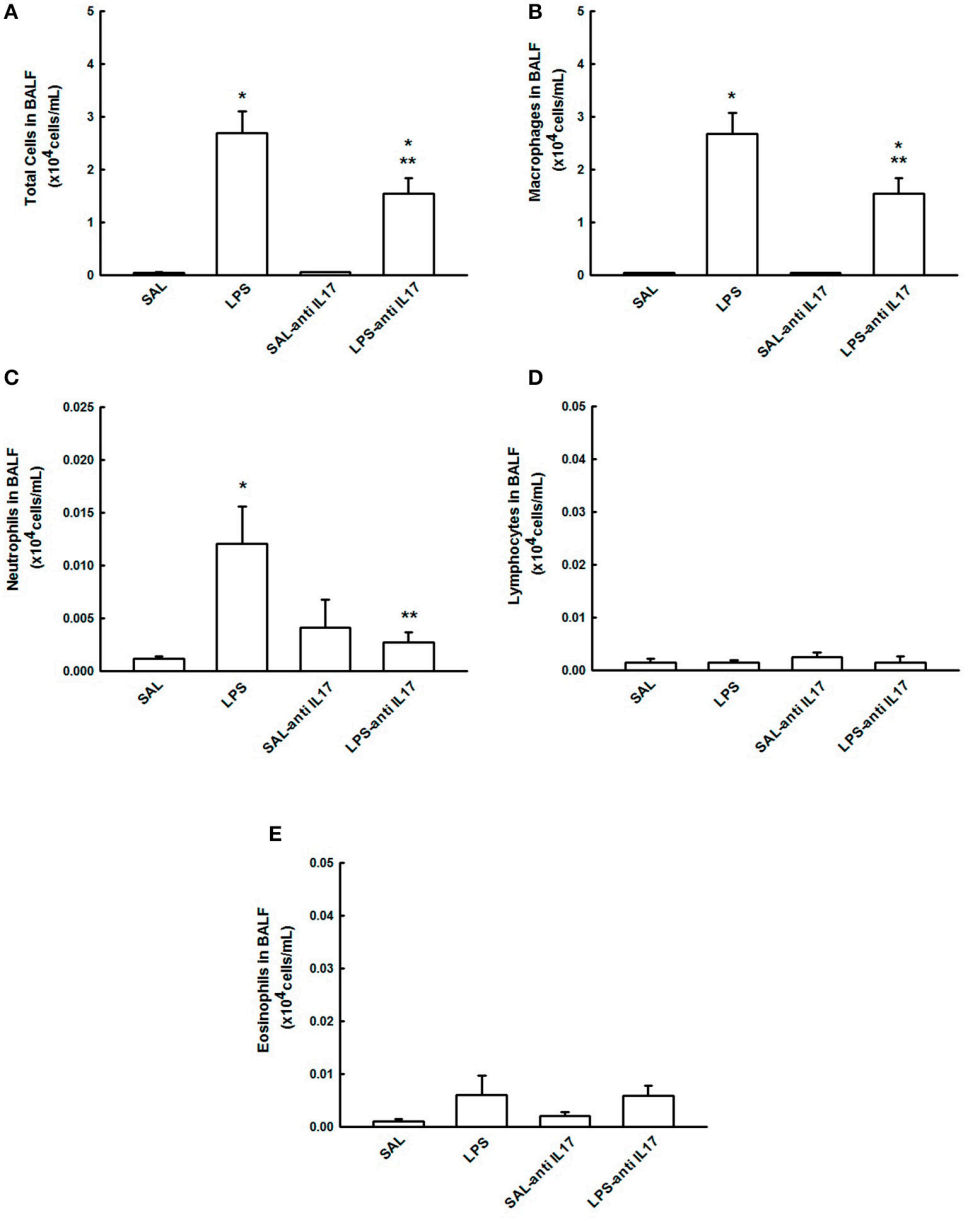
Effects of the pretreatment with anti-IL17 on BALF. **(A)** Total cells, **(B)** cell differential for macrophages, **(C)** cell differential for neutrophils, **(D)** cell differential for Lymphocytes, and **(E)** cell differential for eosinophils. The results are expressed as x10^4^ cells/mL.*P < 0.05 compared to the SAL and SAL-antiIL17 groups; **P < 0.05 compared to the LPS group.

Figure 3 shows the effects of the pretreatment with anti-IL17 on the total inflammatory cells count in the BALF and the differential count for macrophages, neutrophils, lymphocytes and eosinophils.

LPS instillation increased total cell number, and the number of macrophages and neutrophils in BALF compared to the SAL and SAL-antiIL17 groups (*P* < 0.05), which were all decreased by anti-IL17 (*P* < 0.05). There was no effect of LPS or anti-IL17 on the number of lymphocytes and eosinophils in BALF. Anti-IL17 decreased 42.5% of macrophages, 80% of neutrophils, 0% of lymphocytes, and 16.6% of eosinophils compared to the LPS group.

### Alveolar collapse

**Figure 4:**
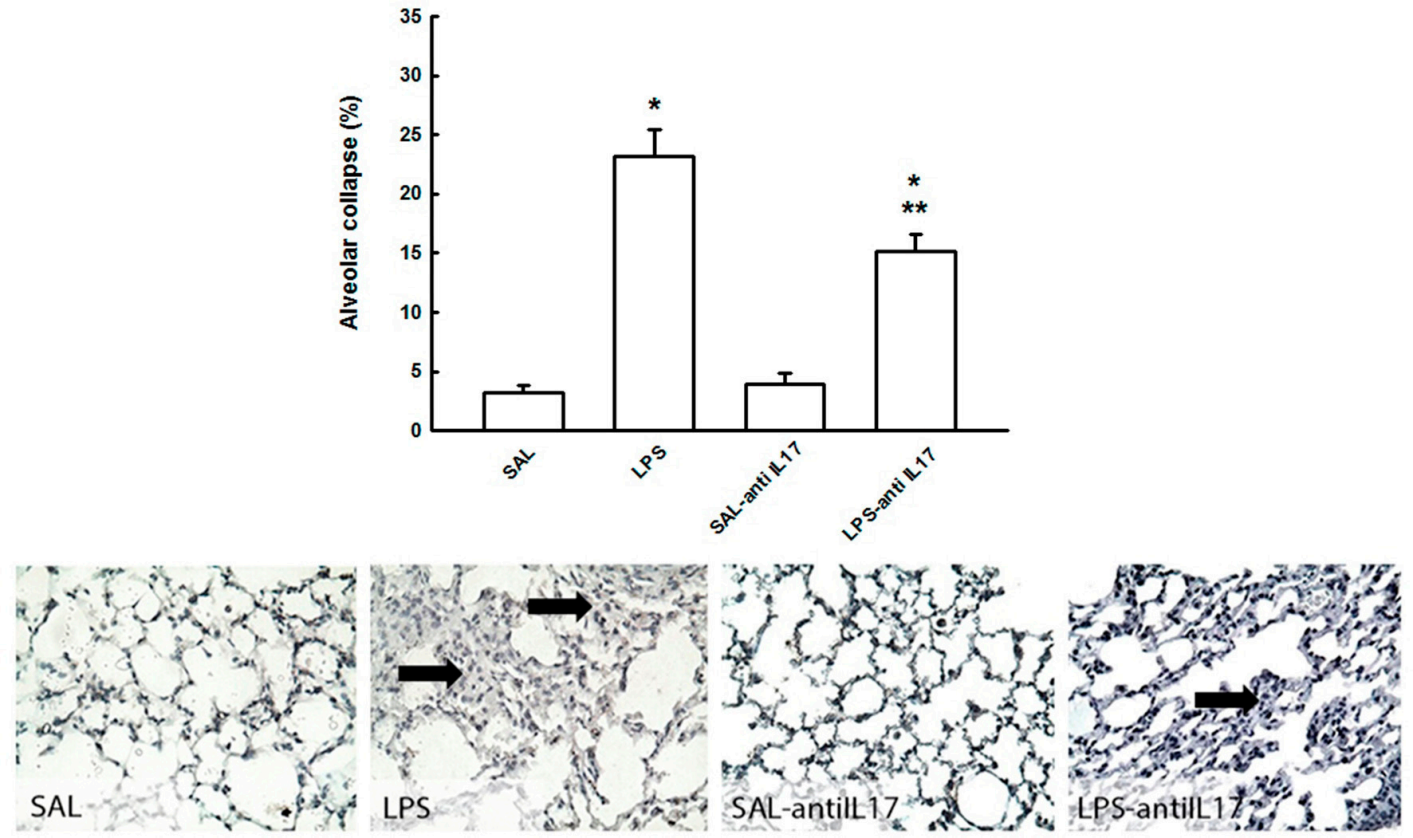
Effects of the pretreatment with anti-IL17 on alveolar collapse (%) and photomicrographs of alveolar collapse. Magnification x1000. Arrow: alveolar collapse. All experimental groups are represented: SAL, LPS, SAL-antiIL17 and LPS-antiIL17 groups. *P < 0.05 compared to the SAL and SAL-antiIL17 groups; **P < 0.05 compared to the LPS group.

Figure 4 shows the effects of the pretreatment with anti-IL17 on the evaluation of alveolar collapse in the four experimental groups. LPS instillation increased in volume fraction of alveolar collapse compared to the SAL and SAL–antiIL17group (*P* < 0.05), which was decreased by anti-IL17 (*P* < 0.05).

### Determination of IL-8 and IL-10 by immunohistochemistry

**Figure 5:**
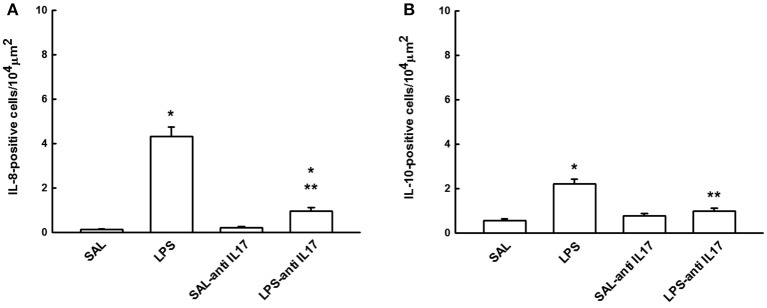
Effects of the pretreatment with anti-IL17 on **(A)** IL-8 and **(B)** IL-10-positive cells in lung tissue. *P < 0.05 compared to the SAL and SAL-antiIL17 groups; **P < 0.05 compared to the LPS group.

Figure 5 shows the effects of the pretreatment with anti-IL17 on the expression of inflammatory markers for IL-8 (A) and IL-10 (B)-positive cells in lung tissue. LPS instillation increased in the number of IL-8 and IL-10-positive cells in lung tissue compared to the SAL and SAL-antiIL17 groups (*P* < 0.05). Anti-IL17 decreased the number of IL-8 and IL-10-positive cells in lung tissue compared to the LPS group (*P* < 0.05).

### Determination of IL-6 and IL-17 by immunohistochemistry and RT-PCR

**Figure 6:**
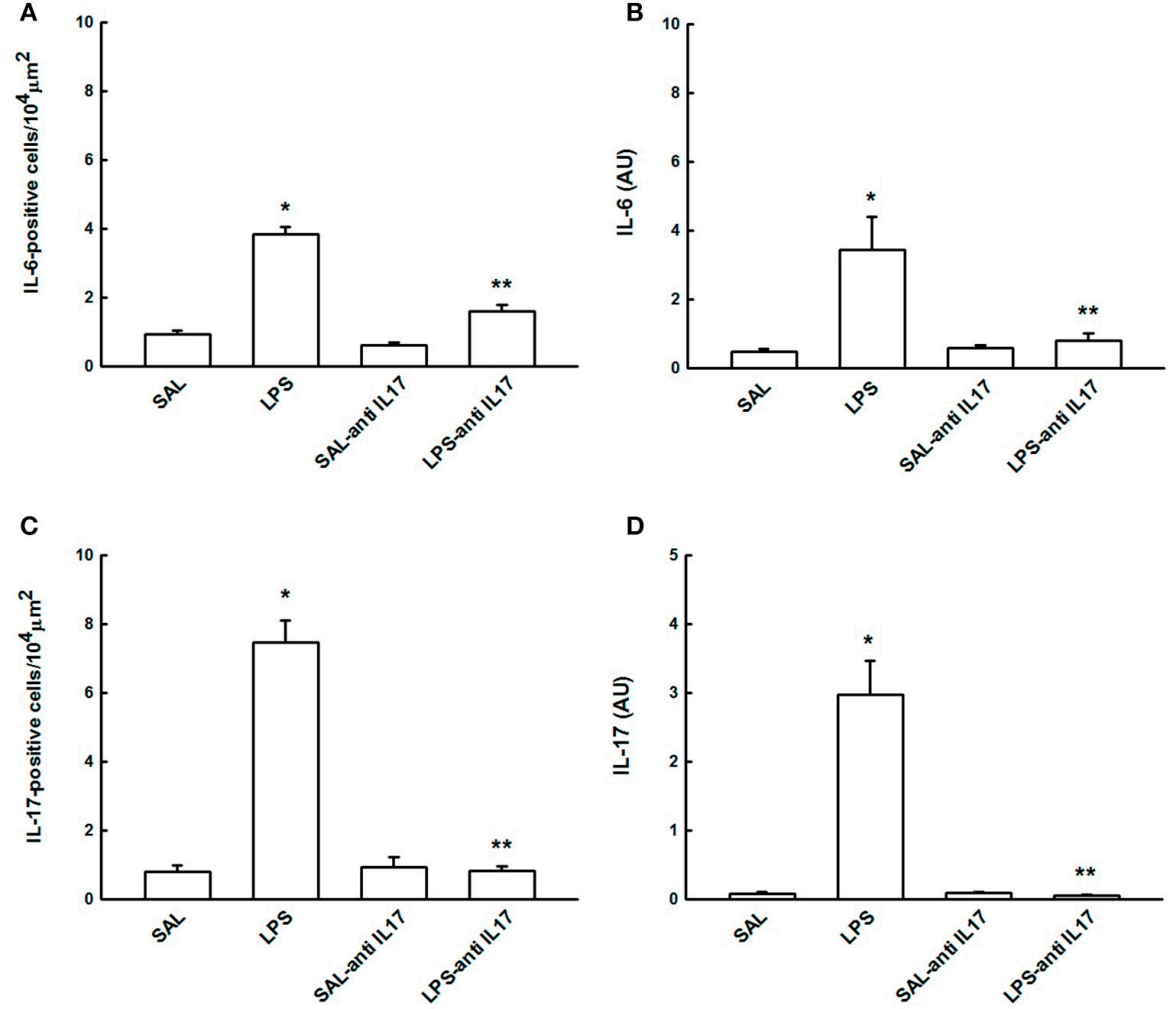
Effects of the pretreatment with anti-IL17 on **(A)** IL-6-positive cells, **(B)** IL-6 gene expression (AU), **(C)** IL-17-positive cells and **(D)** IL-17 gene expression (AU) in lung tissue. *P < 0.05 compared to the SAL and SAL-antiIL17 groups; **P < 0.05 compared to the LPS group.

Figure 6 shows the effects of the pretreatment with anti-IL17 on the IL-6 and IL-17-positive cells and gene expression on lung tissue. LPS instillation increased IL-6 and IL-17-positive cells and gene expression compared to the SAL and SAL-antiIL17 groups (*P* < 0.05). Anti-IL17 decreased the IL-6 and IL-17-positive cells and gene expression compared to the LPS group (*P* < 0.05).

### Determination of TNF-α and IL-1β by ELISA and immunohistochemistry

**Figure 7:**
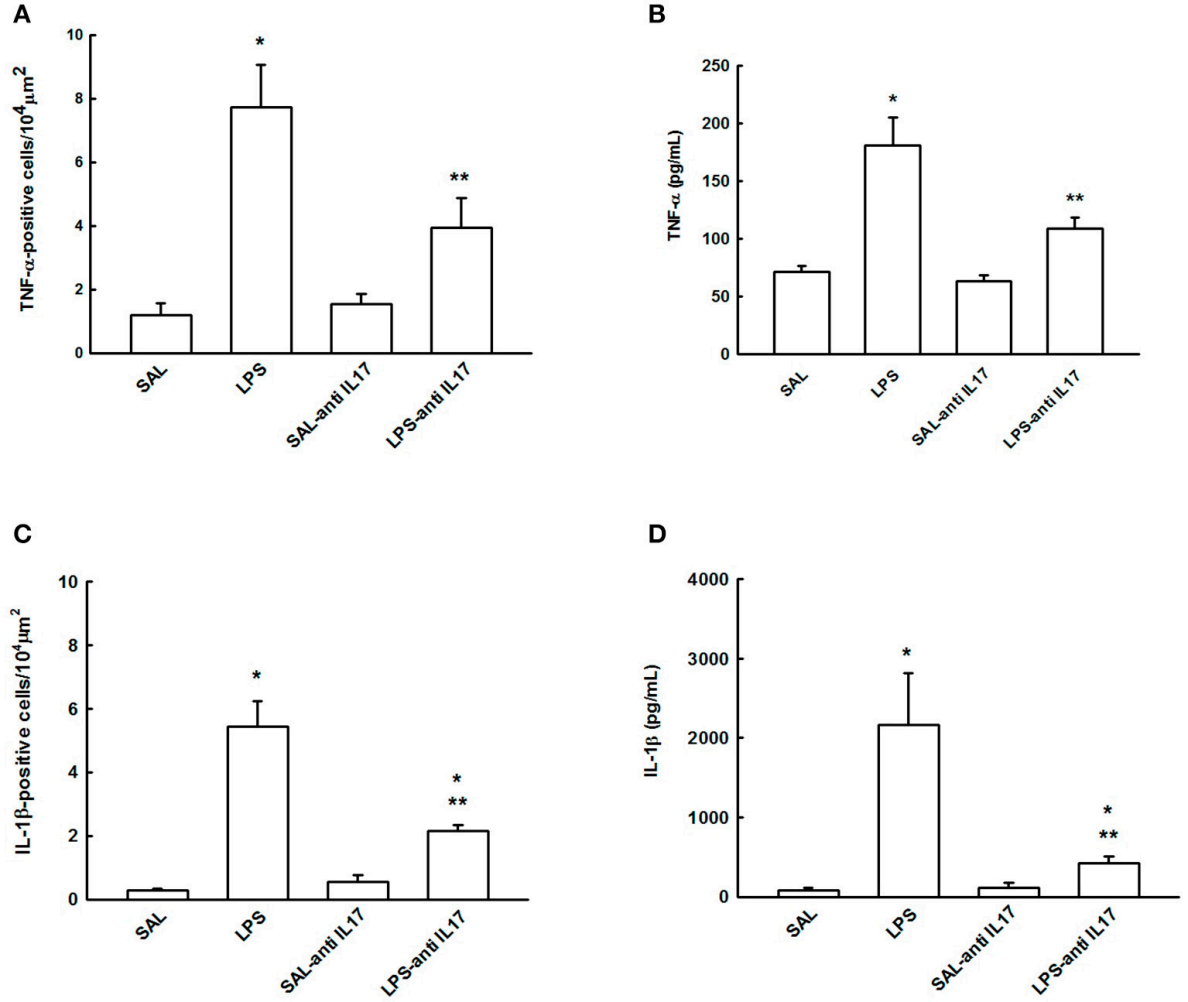
Effects of the pretreatment with anti-IL17 on **(A)** TNF-α-positive cells, **(B)** TNF-α cytokines levels, **(C)** IL-1β-positive cells and **(D)** IL-1β cytokines levels in lung tissue.*P < 0.05 compared to the SAL and SAL-antiIL17 groups; **P < 0.05 compared to the LPS group.

Figure 7 shows the effects of the pretreatment with anti-IL17 on the TNF-α and IL-1β-positive cells (Figures 7A,C) and cytokines levels (Figures 7B,D) on lung tissue. LPS instillation increased the levels of cytokine and positive cells of TNF-α and IL-1β compared to the SAL and SAL-antiIL17 groups (*P* < 0.05). Anti-IL17 decreased the levels of cytokine and positive cells of TNF-α and IL-1β compared to the LPS group (*P* < 0.05).

### CD4+ and FOXP3+ regulatory T cells

**Figure 8:**
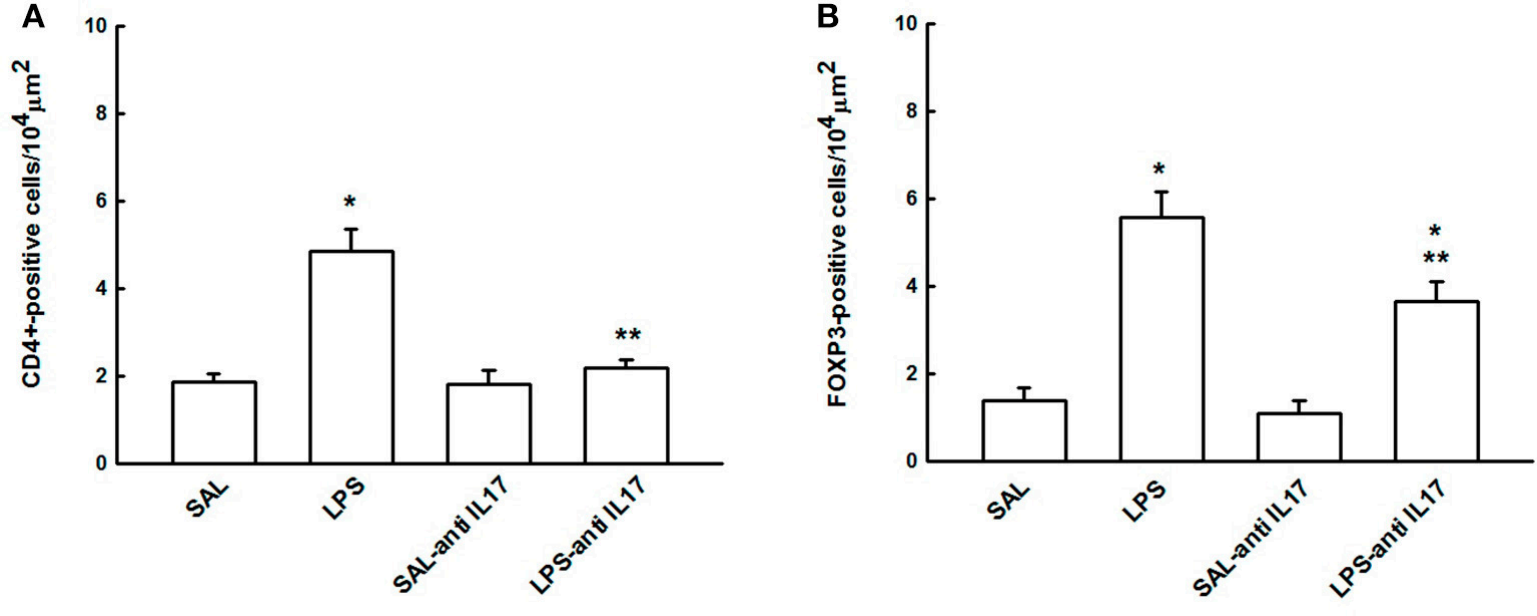
Effects of the pretreatment with anti-IL17 on **(A)** CD4+ and **(B)** FOXP3-positive cells in lung tissue. *P < 0.05 compared to the SAL and SAL-antiIL17 groups; **P < 0.05 compared to the LPS group.

Figure 8 shows the effects of the pretreatment with anti-IL17 on the expression of immune cells regulation CD4+ (Figure 6A) and FOXP3-positive cells (Figure 4B) in lung tissue. We noted that the LPS group presented an increased CD4+ and FOXP3-positive cells compared to the SAL and SAL-antiIL17 groups (*P* < 0.05). Anti-IL17 (LPS-antiIL17group) decreased the CD4+ and FOXP3-positive cells compared to the LPS group (*P* < 0.05). However, LPS-antiIL17 group presented an increased FOXP3-positive cells compared to the SAL and SAL-antiIL17 groups (*P* < 0.05).

### Extracellular matrix remodeling

Table 1 shows the effects of the pretreatment with anti-IL17 on the extracellular matrix remodeling. We observed that the LPS group presented an increased the volume fraction of collagen fibers and actin, and number of TGF-β, MMP-9, MMP-12, and TIMP-1-positive cells compared to the SAL and SAL-antiIL17 groups (*P* < 0.05). Anti-IL17 (LPS-antiIL17group) decreased the volume fraction of collagen fibers and actin, and number of TGF-β, MMP-9, MMP-12, and TIMP-1-positive cells compared to the LPS group (*P* < 0.05). However, LPS-antiIL17 group presented an increased MMP-9 and TIMP-1-positive cells compared to the SAL and SAL-antiIL17 groups (*P* < 0.05) and decreased the volume fraction of collagen fibers and actin compared to the SAL and SAL-antiIL17 groups (*P* < 0.05).

**Table 1:** Absolute values of the morphometric analysis of extracellular matrix remodeling markers in lung tissue.

**Remodelling markers**	**SAL**	**LPS**	**SAL-antiIL17**	**LPS-antiIL17**
Collagen fibers (%)	12.35 ± 1.01	23.90 ± 2.67 ^*^	11.55 ± 0.92	9.65 ± 0.37 ^*^/^**^
Actin (%)	17.92 ± 1.09	27.77 ± 2.38 ^*^	18.75 ± 2.46	9.14 ± 1.91^*^/^**^
MMP-9 (cells/10^4^μm^2^)	2.70 ± 0.46	13.10 ± 0.80 ^*^	1.89 ± 0.38	5.88 ± 0.46 ^*^/^**^
MMP-12 (cells/10^4^μm^2^)	0.32 ± 0.06	4.00 ± 0.22 ^*^	0.32 ± 0.09	0.26 ± 0.11^**^
TIMP-1 (cells/10^4^μm^2^)	1.11 ± 0.18	8.42 ± 0.95 ^*^	1.01 ± 0.25	4.59 ± 0.52 ^*^/^**^
TGF-β (cells/10^4^μm^2^)	1.65 ± 0.23	6.24 ± 0.80 ^*^	1.66 ± 0.25	1.71 ± 0.25^**^

*Collagen fiber and actin are expressed as percentage and MMP-9, MMP-12, TIMP-1 and TGF-β are expressed as the number of positive cells/10^4^μm^2^*.

* P < 0.05, compared to the SAL and SAL-antiIL17 groups;

** *P < 0.05, compared to the LPS group*.

### Oxidative stress response

Table 2 shows the effects of the pretreatment with anti-IL17 on the oxidative stress markers. We noted that the LPS group presented an increased number of iNOS-positive cells, volume fraction of 8-iso-PGF2α and gene expression of ARG-1 compared to the SAL and SAL-antiIL17 groups (*P* < 0.05). Anti-IL17 decreased the number of iNOS-positive cells, volume fraction of 8-iso-PGF2α and gene expression of ARG-1 compared to the LPS group (*P* < 0.05).

**Table 2:** Absolute values of the morphometric analysis of oxidative stress markers in lung tissue.

**Oxidative Stress Markers**	**SAL**	**LPS**	**SAL- antiIL17**	**LPS-antiIL17**
iNOS (cells/10^4^μm^2^)	4.52 ± 0.47	19.60 ± 2.75^*^	6.23 ± 1.24	9.82 ± 1.13^*^/^**^
8-iso-PGF2α (%)	0.36 ± 0.07	2.10 ± 0.21^*^	0.48 ± 0.06	0.19 ± 0.02^**^
ARG-1 (AU)	0.87 ± 0.39	6.54 ± 1.40^*^	1.24 ± 0.47	0.94 ± 0.18^**^

*iNOS is expressed as number of positive cells/10^4^μm^2^. 8-iso-PGF2α is expressed as percentage and gene expression arginase-1 (ARG-1) expressed in arbitrary units (AU)*.

* P < 0.05, compared to the SAL and SAL-antiIL17 groups;

** *P < 0.05, compared to the LPS group*.

### Expression of p65-NFκB

**Figure 9:**
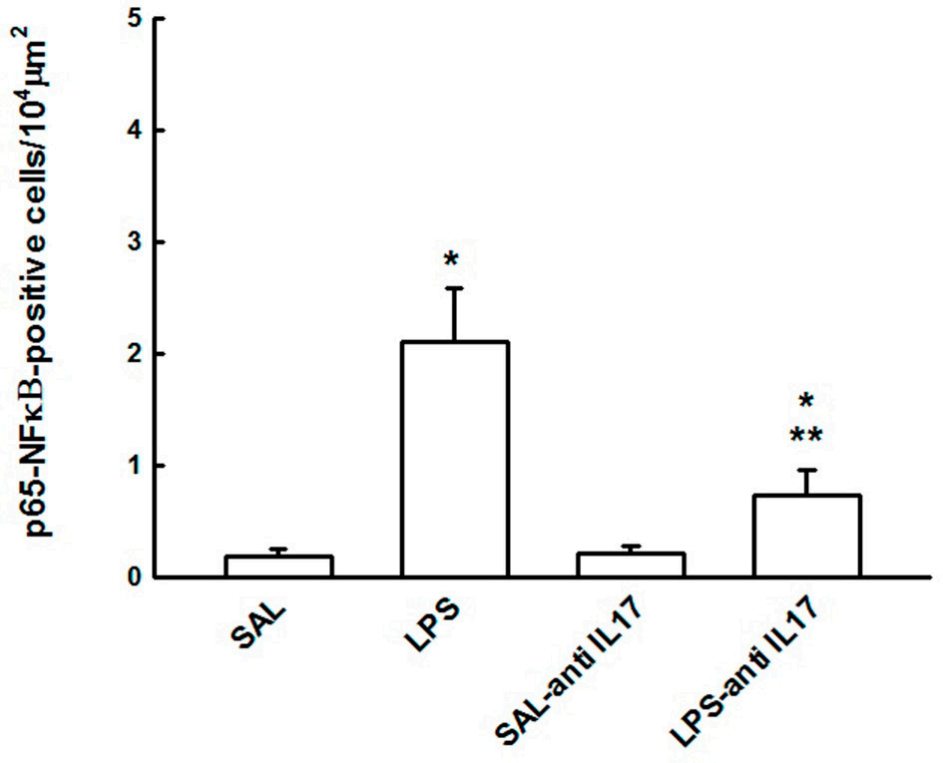
Effects of the pretreatment with anti-IL17 on p65-NFκB-positive cells in lung tissue. *P < 0.05 compared to the SAL and SAL-antiIL17 groups; **P < 0.05 compared with LPS group.

Figure 9 shows the effect of the pretreatment with anti-IL17 on the pro-inflammatory transcription gene factor p65-NFκB in lung tissue. LPS instillation increased in the number of p65-NFκB-positive cells, compared with the SAL and SAL-antiIL17 groups (*P* < 0.05), which was decreased by anti-IL17 (*P* < 0.05).

### Expression of Rock 1 and ROCK 2

**Figure 10:**
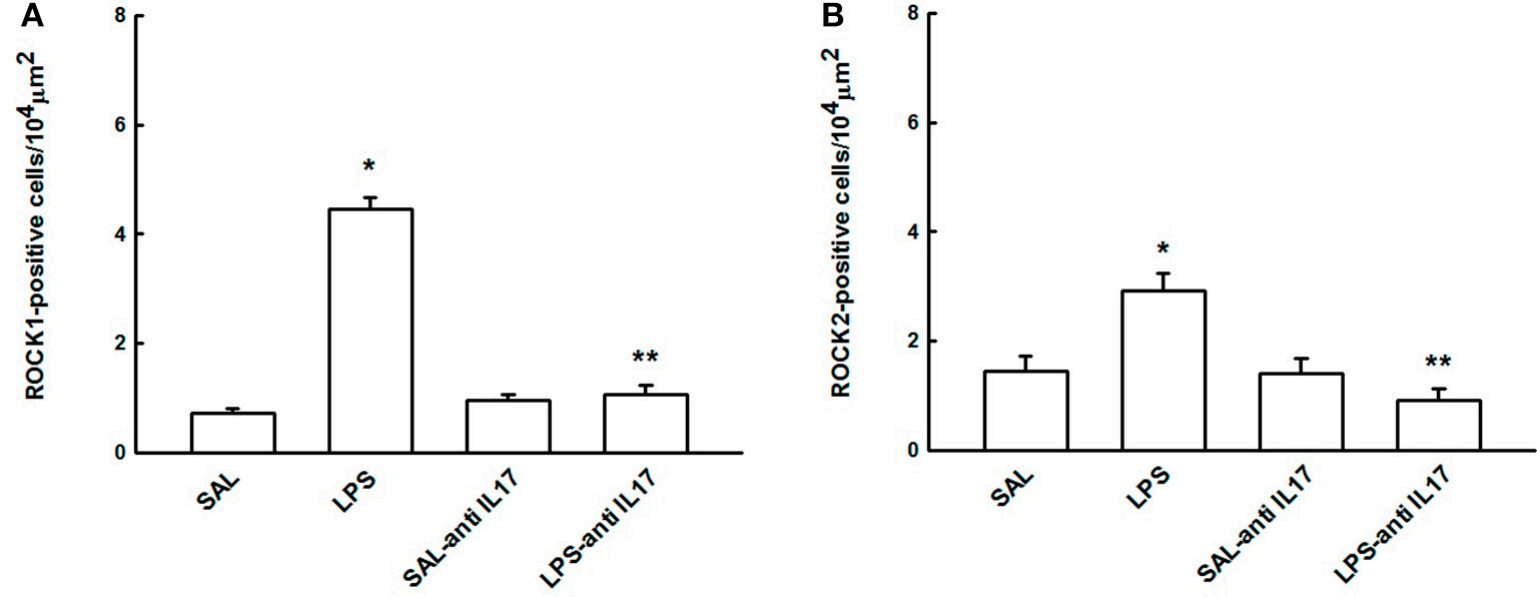
Effects of the pretreatment with anti-IL17 on **(A)** ROCK1 and **(B)** ROCK2-positive cells in lung tissue. *P < 0.05 compared to the SAL and SAL-antiIL17 groups; **P < 0.05 compared to the LPS group.

Figure 10 shows the effects of the pretreatment with anti-IL17 on the expression of ROCK 1(A) and ROCK 2 (B)-positive cells in lung tissue to better understanding the functional lung response observed in the experimental groups. LPS instillation increased the number of ROCK 1 and ROCK 2-positive cells compared to the SAL and SAL-antiIL17 groups (*P* < 0.05), which was decreased by anti-IL17 in both cases (*P* < 0.05).

### Qualitative analysis

**Figure 11:**
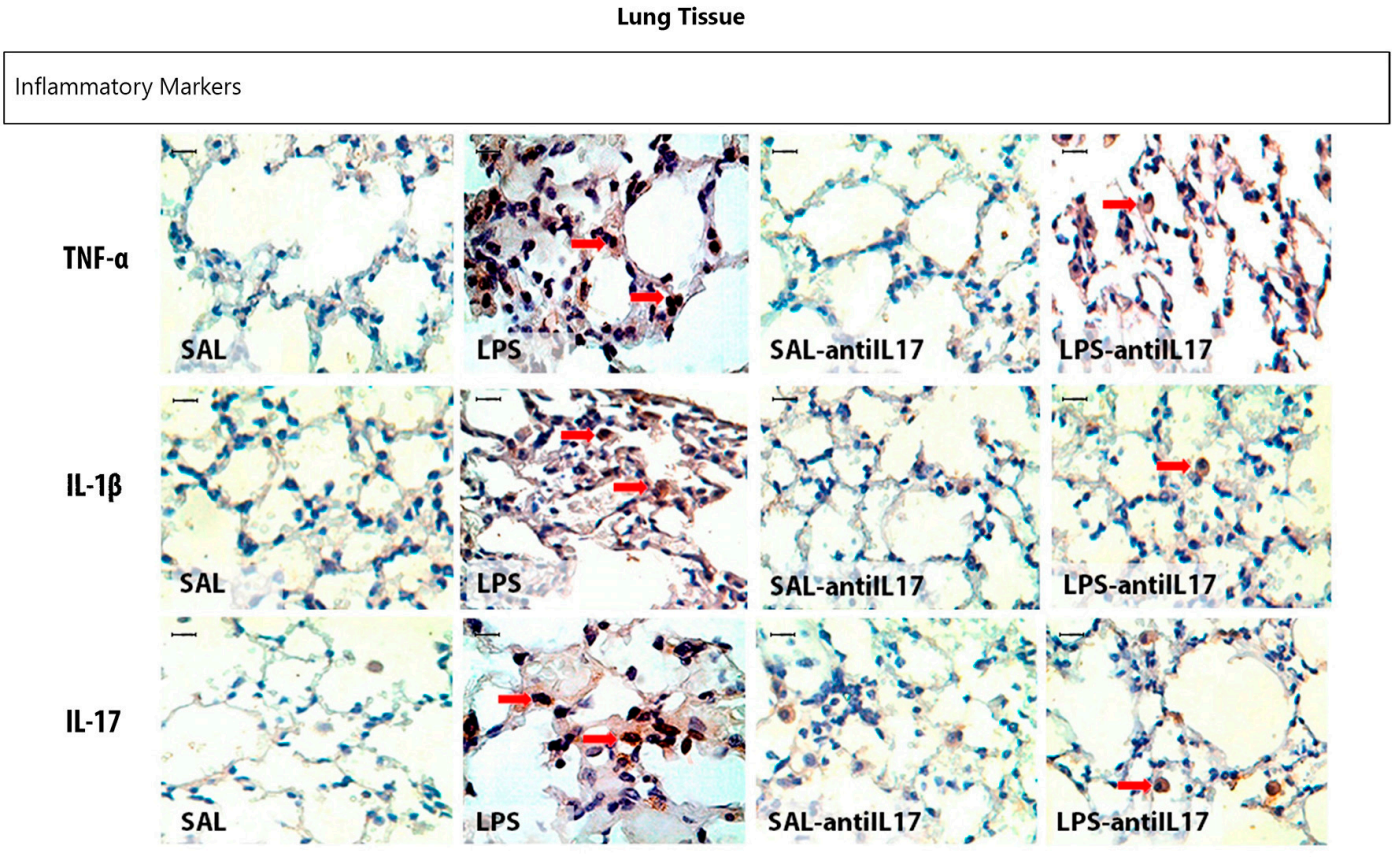
Inflammatory markers in lung tissue: Photomicrographs of TNF-α, IL-1β and IL-17 immunohistochemical stain showing extracellular matrix inflammation in lung tissue. Magnification x1000. All experimental groups are represented: SAL, LPS, SAL-antiIL17, and LPS-antiIL17 groups. Arrow: positive cells. Scale bar = 10 μm.

**Figure 12:**
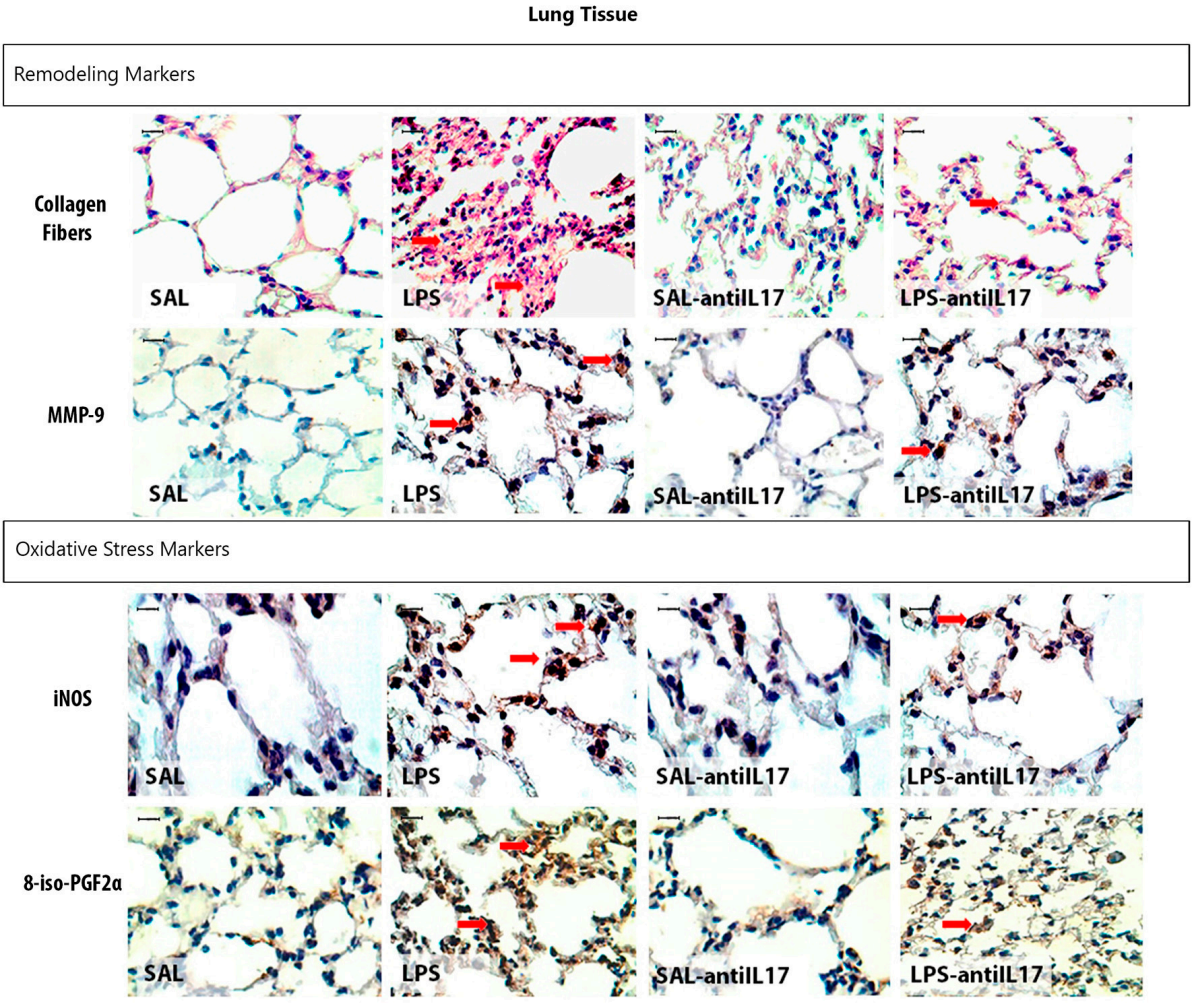
Remodeling and oxidative stress markers in lung tissue: Photomicrographs of immunohistochemical stains showing extracellular matrix remodeling and oxidative stress in lung tissue by detecting collagen fibers, iNOS and 8-iso-PGF2α. Magnification of ×1000. All experimental groups are represented: SAL, LPS, SAL-antiIL17 and LPS-antiIL17 groups. Arrow: positive cells. Scale bar = 10 μm.

Representative photomicrographs are presented in Figure 11, and illustrate the inflammatory processes in the lung tissue. The slides were stained TNF-α, IL-1β, and IL-17-positive cells. Figure 12 illustrates the extracellular matrix remodeling and oxidative stress processes in the lung tissue. The slides were stained for cells expressing collagen fibers, MMP-9, iNOS, and 8-iso-PGF2α. The LPS group showed an increase in the number of TNF-α, IL-1β, IL-17, MMP-9, and iNOS-positive cells and an increase in the volume fraction of 8-iso-PGF2α and collagen fibers, suggesting the presence of lung inflammation, extracellular matrix remodeling, and activation of the oxidative stress processes. Pretreatment with anti-IL17 reduced the above changes in lung tissue, which can be observed in the representative photomicrographs.

## Discussion

This study investigated the effect of and the mechanisms involved in the pretreatment with anti-IL17 in the early phase in a model of ALI induced by LPS. We demonstrated that anti-IL17 pretreatment prior to the intratracheal instillation of LPS decreased pulmonary resistance and elastance, the number of ROCK 1 and ROCK 2-positive cells, eNO concentration, neutrophilic and macrophage lung tissue infiltration, cytokine expression, extracellular matrix remodeling, and oxidative stress responses. In addition, the gene expression of ARG-1, IL-6, and IL-17 was also decreased. These results suggest that blocking the effects of IL-17 can protecting the lung from the inflammatory effects of LPS-induced ALI by altering the inflammatory, extracellular matrix remodeling, and oxidative stress profile in lung tissue.

We also showed that there was a decrease in the resistance and elastance of the respiratory system after anti-IL17. There is evidence that actin/myosin contractility is regulated by Rho-kinase (Sharanek et al., 2016). The influence of Rho-kinase on resistance and elastance of the respiratory system it is related to agonist-mediated Ca^2+^ sensitization, but the mechanisms have not been fully elucidated (Kaneko-Kawano et al., 2012; Pigati et al., 2015). Nguyen et al. (2013) evaluated IL-17 and Rho-kinase in endothelial dysfunction and hypertension and in agreement with our study, showed that IL-17 caused a threefold increase in the Rho-kinase activator RhoA, which was prevented by an IL-17 neutralizing antibody.

In this study, we also demonstrated a reduction in the number of neutrophils and macrophages in BALF. Even though neutrophils are the hallmark effector cells of acute inflammation, they also contribute to chronic inflammation and adaptive immune responses via IL-17. IL-17 secreted from neutrophils induces the release of pro-inflammatory factors (such as cytokines, chemokines and metalloproteinases) from mesenchymal and myeloid cells, leading to the further neutrophil recruitment and activation (Abraham, 2003; Ley et al., 2007; Caucheteux et al., 2017).

Cells of monocyte-macrophage lineage are another important source of and responders to IL-17 (Ge et al., 2014). In genetically modified mice IL-17 receptor A (IL-17Ra) affects monocyte homeostasis and the kidney fibrotic response to injury (Ge et al., 2014) while preventing macrophages apoptosis and increasing their response to IL-10 and glucocorticoids (Zizzo and Cohen, 2013). In another study, IL-17 elicited a distinct transcriptional profile in mouse macrophages and contributed to monocyte attraction and adherence, while anti- IL-17 antibodies prevented atherosclerotic lesion progression (Erbel et al., 2014). In agreement with these studies, we showed that a reduction of IL-17 caused a decrease in neutrophil and macrophage cellular influx in BALF, reinforcing the importance of IL-17 signaling in neutrophilic and macrophage inflammatory processes. This inhibition with anti-IL17 was able to protect lung tissue from the inflammatory process and reduce areas of collapse.

Macrophages and neutrophils are the first line of defense and are essential for the control of common bacterial infections and provide the costimulatory and cytokine signals to promote T-cell differentiation (Witter et al., 2016). This process is essential for the differentiation process of Th17 cells and is stimulated by the activated cytokines STAT3 IL-6, IL-21 and IL-23, along with IL-1β and TGF-β (Xu and Littman, 2018). IL-6 is mainly produced by the innate immune system and is one of the first cytokines released in the acute phase of ARDS/ALI and is followed by increases in the expression of IL-1β, IL-8, TNF-α, and IL-10 (Blondonnet et al., 2016). During the initiation of Th17 cell development, IL-6 stimulates IL-21 in the early activated CD4 + T cells, thereby serving as a positive amplification loop to induce Th17 cell differentiation (Huang et al., 2012). Both IL-6 and IL-1β enhance the generation of Th17 but in contrast to this, elevated levels of TGF-β induce the differentiation of regulatory T (Treg) cells (Xu and Littman, 2018). Zhao et al. (2017) shows that Th17 inhibition with curcumin treatment was associated with a decrease in CD4 + T cell proliferation and IL-6, IL-23, IL-17A, and RORγt production. In this current study, we observed that anti-IL17 treatment inhibited Th17 cell differentiation by reducing the expression of IL-6 and TGF-β. It was demonstrated that anti-IL17 influenced both the initiation and the stabilization stages of Th17 cell differentiation.

Regarding oxidative stress, we observed a decrease in eNO, iNOS, and 8-iso-PGF2α in the lung tissue in the LPS-antiIL17 group compared to LPS group. A few studies have assessed the effects of nitric oxide synthase inhibition in modulating the inflammatory and functional responses that occur in the lung tissue (Righetti et al., 2014; Pigati et al., 2015; Camargo et al., 2018). Aristoteles et al. (2013) also found a reduction in iNOS positive cells and arginase after treatment with a specific iNOS inhibitor (1400W) and L-NAME in guinea pigs with chronic lung inflammation. Camargo et al. (2018) showed that anti-IL17 reduced iNOS-positive cells in lung tissue in a mice model of chronic allergic inflammation exacerbated induced by LPS.

It has been clearly demonstrated that iNOS activation contributes to the promotion of nitric oxide and peroxynitrite production, which leads to lipid peroxidation and 8-iso-PGF2α generation (Prado et al., 2011; Theodoro-Júnior et al., 2017). 8-iso-PGF2α generation contributes to smooth muscle contraction via tyrosine kinases and Rho/Rho-kinase, leading to the decreased activity of myosin light-chain phosphatase and increasing the level of phosphorylated myosin light chain and contraction (Righetti et al., 2014; Pigati et al., 2015). Moreover, concentrations of 8-iso-PGF2α induced a dose-dependent increase monocyte–endothelial, neutrophils recruitment and calcium release from intracellular stores (Leitinger et al., 2001). In addition, treatment of human macrophages with 8-iso-PGF2α resulted in increase in the expression of IL-8, important interleukin involved in differentiation of Th-17 and polymorphonuclear cells infiltration (Scholz et al., 2003; Gasch et al., 2014; French et al., 2018).

There have been no previous studies analyzing the effects of anti-IL17 on oxidative stress in acute lung injury, but we speculate that the reduction in macrophages, TNF-α, IL-1β, and IL-17-positive cells seen in our study following IL-17 treatment could be responsible the reduction in oxidative stress, as demonstrated in other studies (Bittencourt-Mernak et al., 2017; Theodoro-Júnior et al., 2017; Camargo et al., 2018).

We assessed extracellular matrix remodeling primarily by looking at changes in volume fractions of collagen fibers, where we observed a significant reduction following anti-IL17 treatment. Acute lung inflammation is also associated with the up-regulation of MMP-9 and MMP-12, which are secretory products of activated neutrophils and macrophages (Hergrueter et al., 2011; Hsu et al., 2016). High levels of these two MMPs in BALF fluid of individuals with ALI could reflect effects of pro-inflammatory stimuli on resident alveolar macrophages and newly recruited neutrophil (Grommes and Soehnlein, 2011). In some cases, it can be directly produced by parenchymal cells in response to proinflammatory products such as bacterial LPS or immune complexes (Rosadini and Kagan, 2017). Prause et al. (2004) showed *in vivo*, that the intranasal stimulation of mice with IL-17 induced increases the concentration of biologically active MMP-9 as well as its precursor molecule in airways. In the current study, we also observed that there was augmentation of TIMP-1 in the lung parenchyma after LPS instillation. It is important to note that the specific tissue inhibitor of MMP-9, TIMP-1, has been recognized as having biologic effects independent of MMPs, such as inducing accelerate lung function decline and cellular apoptosis (Barnes, 2016; Lo et al., 2018). These results are in accordance with the idea that a process of degradation/turnover of collagen fibers in the lung parenchyma is continuously occurring, which is induced by inflammatory stimuli, as previously suggested in other studies (Righetti et al., 2014; Bittencourt-Mernak et al., 2017; Hendrix and Kheradmand, 2017). In the current study, the reduction of MMP-9, MMP-12, and TIMP-1-positive cells with anti-IL17 is associated with the reduction of the IL-17-mediated inflammatory process including the recruitment of neutrophils and macrophages.

NFκB is well characterized regulator of the expression of pro-inflammatory cytokines, including TNF-α, IL-6, and IL-1β (Li et al., 2017; Rosadini and Kagan, 2017). Li et al. (2017) showed that anti-IL17 was effectively inhibited LPS-induced expression and activation of pERK1/2 and p65-NFκB in lung tissues and suppressed nuclear translocation of p65-NFκB. Overall, our results indicated that anti-IL17 potently inhibited LPS-activated NFκB signaling in the lung tissue of ALI mice model, leading to significant suppression of inflammation and tissue injury.

Li et al. (2017) and Ding et al. (2017) evaluated the role of anti-IL-17 treatment in the activation of NFκB and inflammation in acute lung injury induced by LPS, respectively. It is important to emphasize that the our study complements the understanding of the pathophysiological process, showing the activation of signaling pathways of changes in lung functions and inflammation with the cell expression of ROCK 1, ROCK 2, CD4+, FOXP3, aspects of the extracellular matrix remodeling such as the evaluation of MMP-9, MMP-12 and TIMP-1 and cell expression as well as several markers of oxidative stress pathway activation (arginase 1, exhaled nitric oxide, iNOS and 8-iso-PGF2α).

The present study has some limitations. IL-17 was inhibited with a monoclonal antibody to IL17 in an experimental model of acute lung injury and we cannot extrapolate our findings directly to human beings. Another important point, we used a single dose of anti-IL17 prior to LPS instillation in order to clarify the role of IL-17 in the pathophysiology of acute lung injury. However, in order to determine whether anti-IL17 could be used as a potential therapeutic strategy, the treatment would need to be given following the exposure to LPS, with dosing regimens that need to be determined and including long term outcomes, such as survival. The strongly of our studies supported the importance of anti-IL-17 in the modulation of the respiratory mechanics, lung inflammatory responses, extracellular matrix remodeling, and oxidative stress pathways activation in an experimental model of acute lung injury. In addition, our results also added the importance of the control of ROCK 1, ROCK 2, and NFκB expression in these responses. Additional studies are needed to elucidate other mechanisms responsible for these changes.

## Conclusion

In conclusion, our results show that pretreatment with anti-IL17 plays an important role in protecting the lung from the inflammatory, remodeling and oxidative stress effects of LPS-induced ALI. The effects of anti-IL17 are mainly mediated by the reduction in expression of inflammatory cytokines, neutrophils and macrophages, suggesting that anti-IL17 can modulate the inflammatory, extracellular matrix remodeling and the oxidative stress balance in ALI. This suggests that further studies using anti-IL17 in a treatment regime would be highly worthwhile.

## Availability of data and material

The datasets used and analyzed during the present study are available from the corresponding author on reasonable request.

## Author contributions

IT, MM, CP, and RR conceived and designed the experiments. EL, MC, BS-R, MA-V, RR, SF, FdS, FS, MA, IG, CP, TS, LC, and LA performed the experiments. RR, MC, CP, IG, EL, and IT analyzed the data. RR, IT, MM, EL, and MA-V contributed reagents, materials, analysis tools. IT and RR wrote the paper. IG assisted in the execution of the experiments involved in the responses to the reviewers. FS, SF, and MC performed the experiments.

### Conflict of interest statement

The authors declare that the research was conducted in the absence of any commercial or financial relationships that could be construed as a potential conflict of interest.

## Abbreviations

ALI: acute lung injury
ARDS: acute respiratory distress syndrome
BALF: bronchoalveolar lavage fluid
BSA: bovine serum albumin
eNO: exhaled nitric oxide
Ers: respiratory elastance
Rrs: respiratory resistance
MMP-9: Metalloproteinase 9
MMP-12: Metalloproteinase 12
TIMP1: Tissue inhibitor of metalloproteinase-1
ROCK: Rho-kinase
iNOS: inducible nitric oxide synthase
TNF-α: Tumor necrosis factor – α
8-iso-PGF2α: 8-isoprostane-PGF2α
IL: interleukin
NF: nuclear factor
